# Study on the pathogenesis of MiR-6324 regulating diarrheal irritable bowel syndrome and bioinformatics analysis

**DOI:** 10.3389/fphar.2023.1044330

**Published:** 2023-02-15

**Authors:** Jin Xiao, Yan-ni Zhou, Yan-lin Yang, Li He, Ke-kai Wang, Min Chen

**Affiliations:** ^1^ Hospital of Chengdu University of Traditional Chinese Medicine, Chengdu, Sichuan, China; ^2^ Sichuan Hospital of Integrative Medicine TCM, Chengdu, Sichuan, China; ^3^ Zigong Fifth People’s Hospital, Zigong, Sichuan, China; ^4^ Traditional Chinese Medicine Hospital, Chongqing, China

**Keywords:** miR-6324, irritable bowel syndrome, bioinformatics analysis, pathogenesis, R language

## Abstract

**Objective:** To investigate the pathogenesis of IBS-D by bioinformatics analysis of the differential microRNAs in rat colon tissue and to analyze and predict the function of their target genes.

**Methods:** Twenty male Wistar rats of SPF class were randomly divided into two groups, the model group was manipulated using the colorectal dilatation method + chronic restraint stress method to establish the IBS-D model; while the blank group stroked the perineum at the same frequency. Screening of differential miRNAs after High-throughput sequencing of rat colon tissue. GO and KEGG analysis of target genes using the DAVID website, further mapping using RStudio software; the STRING database and the Cytoscape software were used to obtain the protein interaction network (PPI) of the target genes as well as the core genes. Finally, qPCR was used to detect the expression of target genes in the colon tissue of two groups of rats.

**Results:** After the screening, miR-6324 was obtained as the key of this study. The GO analysis of target genes of miR-6324 is mainly involved in protein phosphorylation, positive regulation of cell proliferation, and intracellular signal transduction; it affects a variety of cellular components such as cytoplasm, nucleus, and organelles on the intracellular surface; it is also involved in molecular functions such as protein binding, ATP binding, and DNA binding. KEGG analysis showed that the intersecting target genes were mainly enriched in cancer pathways, proteoglycans in cancer, neurotrophic signaling pathway, *etc.* The protein-protein interaction network screened out the core genes mainly Ube2k, Rnf41, Cblb, Nek2, Nde1, Cep131, Tgfb2, Qsox1, and Tmsb4x. The qPCR results showed that the expression of miR-6324 decreased in the model group, but the decrease was not significant.

**Conclusion:** miR-6324 may be involved in the pathogenesis of IBS-D as a potential biological target and provide further ideas for research on the pathogenesis of the disease or treatment options.

## 1 Introduction

Irritable bowel syndrome (IBS) is a functional enteropathy with abdominal pain, bloating, or abdominal discomfort as the main symptoms, lacking organic lesions of structural and biochemical abnormalities of the gastrointestinal tract (Li et al., 2018). Irritable bowel syndrome can be divided into four subtypes, which can be classified according to the Rome IV criteria into the following four types: 1) Constipated IBS with constipation as the main symptom (IBS-C) 2) diarrheal IBS with diarrhea as the main symptom (IBS-D) 3) Mixed IBS with alternating diarrhea and constipation as the main symptom (IBS-M) 4) indeterminate IBS with stool characteristics that do not meet any of the criteria in the appeal (IBS-U). The most common of which is irritable bowel syndrome with diarrhea and abdominal pain as the main manifestation (IBS-D) (Ha and Kim, 2014). The prevention and treatment of IBS is an important public health task, as it has a significant impact on the normal life as well as the psychological health of patients with irritable bowel syndrome. miRNAs have potential gene regulatory functions (Ha and Kim, 2014), and their main function is to bind to the complementary 3′UTR of the target messenger mRNA, reducing its stability as well as inhibiting translation (Mansour et al., 2016). Research on the pathogenesis of IBS-D is still unclear and is associated with multiple causes, but the main causes currently agreed by researchers are the following: alterations in the intestinal flora (Hou et al., 2022), abnormalities in the brain-gut axis (Wu et al., 2022), emotional abnormalities such as depression and anxiety (Chen et al., 2021), and intestinal barrier dysfunction (Hanning et al., 2021). miRNA has been closely linked to the development and treatment of several human diseases, mostly for cancer-related studies, and because of its widespread presence in the human body, more non-cancerous diseases have also been shown to be associated with it. So, We believe that some miRNAs can be studied and explored as therapeutic targets for IBS-D. At present, the number of studies using animal experiments combined with bioinformatics to explore the pathogenesis of IBS-D is relatively small. In this study, we propose to conduct bioinformatics analysis to screen the core genes and important pathways for miR-6324 in rat colon and provide a more reliable basis for the diagnosis and treatment of IBS-D.

## 2 Materials and methods

### 2.1 Experimental animals and feeding environment

Twenty male Wistar rats of SPF grade 5–6 weeks old weighing 160–200 g were randomly and equally assigned into blank control group and model group, 10 rats per group. The rats were housed in standard class II animal rooms, quiet and noiseless, with circulating ventilation in the experimental environment. The room temperature was 22°C ± 2°C, the room humidity was 50% ± 10%, and the light-to-dark ratio was 1/1. After 1 week of acclimatization feeding. The rats were housed in the Animal Experiment Center of Chengdu University of Traditional Chinese Medicine in a quiet environment with suitable temperature and humidity during the experiment. The experiments were performed daily at 8–10 a.m. The experimental animals were provided by Chengdu Dashuo Experimental Animal Co., Ltd. with animal production license No. SYXK (Chuan) 2015-196. All animal studies were approved by the University Institutional Review Board and the Institutional Animal Care and Use Committee, respectively. Ethical review number: 2020DL-001.

### 2.2 Instruments and reagents

#### 2.2.1 Instruments

Centrifuge 5418R benchtop centrifuge (Eppendorf, Germany); Nano Drop 2000 UV spectrophotometer (Thermo, United States); Qubit2.0 quantification instrument; EI0001 electrophoresis system (Invitrogen, United States) D195M fluorescence transilluminator (Clare Chemical Research, United States); 2100 biochip analyzer (Agilent, United States); MyCycler PCR (Bio-rad, United States); Fluorescence quantitative PCR instrument (Roche, Switzerland).

#### 2.2.2 Reagents

TruSeq Stranded Total RNA with Ribo-Zero Gold (Illumina, United States); AgencourtAMPure XP (BECKMAN COULTER, United States); Agencourt RNAClean XP(BECKMAN COULTER, United States); QubitRNA Assay Kit (Life Technologies, United States); QubitdsDNA Assay Kit (Life Technologies, United States); Bioanalyzer 2100 RNA-6000 Nano Kit (Aglient, United States); Bioanalyzer 2100 DNA-1000 Kit (Agilent, United States); SuperScript II Reverse Transcriptase (Invitrogen, United States); miRNA first strand cDNA synthesis kit (Accurate Biology, China); TRIpure Reagent Total RNA Extraction Reagent (Bioteke Corporation, China); primer (Tsingke Biotechnology Co., China).

### 2.3 Model Preparation

The IBS-D animal model was established using a combination of the colorectal dilatation method + chronic restraint stress method in the model rats. The rats were gently touched on the anal region before the experiment to induce them to evacuate feces. The homemade colorectal dilatation balloon coated with paraffin oil was gently inserted into the rectum of the rat about 7 cm, and the catheter was fixed at the tail of the rat, about 1 cm from the anus, after which the rat was fixed in a transparent observation chamber (20*6*8 cm) so that it could only move back and forth, and the colorectal dilatation was performed after waiting for the rat to calm down, and the dilatation pressure was 80 mmHg once/day for 2 weeks; meanwhile, the blank control group only stroked the perineum with the same frequency.

### 2.4 Abdominal wall retraction reflex score (AWR score)

At the end of modeling, an AWR test was performed and scores were recorded for both groups of rats. The performance of the rats was recorded and scored at 20, 40, 60, and 80 mmHg pressures, at a frequency of 20 s/time, with a total of three dilations, each at an interval of 5 min, and the average of the three times was taken as a backup. The AWR scoring criteria are shown in Table 1. At the end of the experiment, the rats were anesthetized with 2% sodium pentobarbital intraperitoneally at a dose of 2.5 ml/kg. The skin and subcutaneous tissues and the greater omentum were cut along the midline of the rat’s abdomen using scissors, and the terminal tissues of the colon and rectum were exposed and separated, and the tissues of the intestinal segment 6 cm from the end of the anus were removed for the next step of testing. After sampling, the rats were euthanized using decapitation.

**TABLE 1:** AWR scoring criteria.

Score	Performance
0	No behavioral response in rats
1	The rat’s body was stationary and only the head moved
2	Contraction of abdominal muscles in rats
3	Contraction and lifting of abdominal muscles in rats
4	The abdominal muscles of rats contract strongly and lift the pelvis and perineum

### 2.5 High-throughput sequencing

After the end of modeling, three rats from each group are randomly selected for the next step. Rat colon tissue was taken and preserved, total RNA was extracted and processed for PCR amplification, and the constructed libraries were quality-checked with Agilent 2100 Bioanalyzer after passing. The libraries were sequenced using the Illumina sequencer, and the differential miRNAs with *p*-value < 0.05 and FC = 2-fold were taken as a backup.

### 2.6 KEGG pathway analysis and GO analysis

Two target gene prediction software, miRWalk 3.0 (http://mirwalk.umm.uni-heidelberg.de); Targetscan 7.2 (http://targetscan.org/vert_72/), were used for target gene prediction and the valid data intersection of the two databases was taken and retained. The miRNAs containing the neurotrophin signaling pathway in the KEGG pathway were screened using DAVID 6.8 (http://david.ncifcrf.gov/) and kept for backup, and gene function enrichment analysis and mapping were performed using RStudio software.

### 2.7 Protein-protein interaction (PPI) analysis

Target genes were analyzed using STRING 11.0 (https://string-db.org/) and then the network relationships were mapped using Cytoscape.

### 2.8 qPCR for miRNA

First, RNA was extracted from colon tissue using TRIpure Reagent Total RNA Extraction Reagent, then miRNA first strand cDNA synthesis kit was added according to the kit instructions, and the RNA was reverse transcribed to cDNA using MyCycler PCR set at 37°C for 60 min and 85°C for 5 min. Finally, primers were added and the initial Ct value was obtained by fluorescence quantitative PCR instrument for miR-6324. The expression level of MiR-6324 was calculated by ΔCt method (2^-Δ Δ Ct method).

Primers: F-TCAGTAGGCCAGACAGCAAG.

### 2.9 Data Processing

SPSS26.0 software was used to analyze the data, and the data obtained in this experiment were all measures. AWR score data met normal distribution with homogeneous variance, and *t*-test was used for analysis; the PCR test data conforms to the normal distribution, but the variance is uneven, the data were statistically analyzed using t’ test. A *p*-value <0.05 was considered statistically significant.

## 3 Result

### 3.1 Abdominal wall retraction reflex score (AWR score)

After modeling, the AWR scores were significantly higher in the model group at 20 mmHg, 40 mmHg, and 60 mmHg compared with the blank group at the same pressure (*p* < 0.05); at 80 mmHg pressure, the difference in AWR scores between the blank and model groups was not significant *p* > 0.05), as shown in Table 2, and the above results suggest that the modeling was successful.

**TABLE 2:** Comparison of AWR scores between the two groups after modeling (‾X ± S).

Group	20mmHg	40mmHg	60mmHg	80mmHg
Blank group	0.12 ± 0.170	1.62 ± 0.485	2.29 ± 0.627	3.50 ± 0.436
Model group	1.54 ± 0.776*	3.33 ± 0.356*	3.71 ± 0.330*	3.87 ± 0.353

Note: The symbol * indicates *p* < 0.05 compared with the blank group at the same pressure. Under the pressure of 20/40/60 mmHg, the scores of the model group were all significantly higher than those of the blank group; while under the pressure of 80 mmHg, there was no significant difference in the scores of the rats in the model and blank groups. This indicates that the visceral sense of the rats in the model group was higher than that of the blank group under 20/40/60 mmHg pressure; while under 80 mmHg pressure, the scores of the model group and the blank group converged and the differences disappeared.

### 3.2 High-throughput sequencing

**FIGURE 1:**
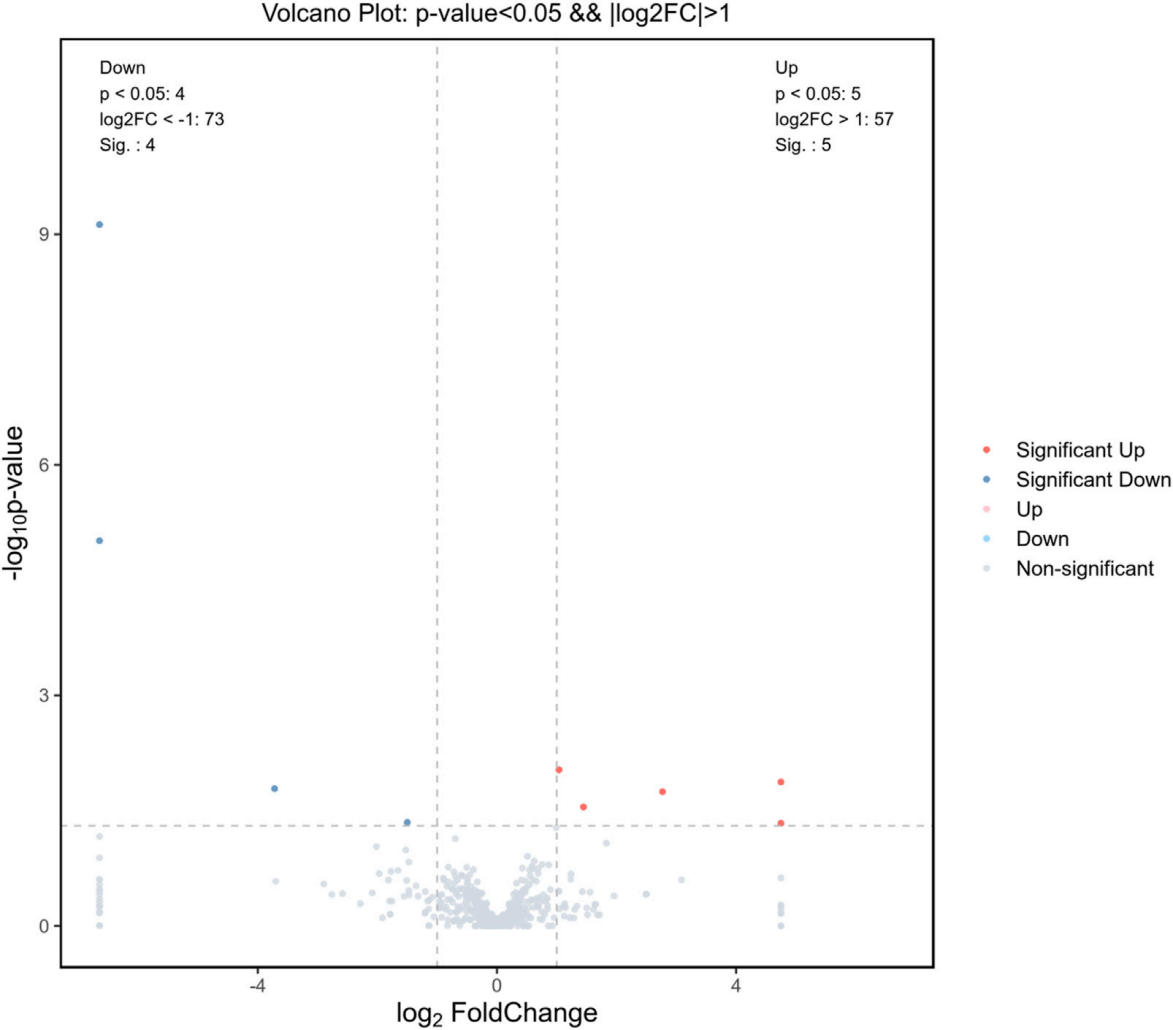
Differential genetic volcano map. Gray indicates no difference, red and blue indicate genes with significant differences (*p* < 0.05 and ∣log2FC∣ > 1), nine in total. The left side (blue dots) indicates genes with downregulated expression, four in total; and the right side (red dots) indicates genes with upregulated expression, five in total.

**FIGURE 2:**
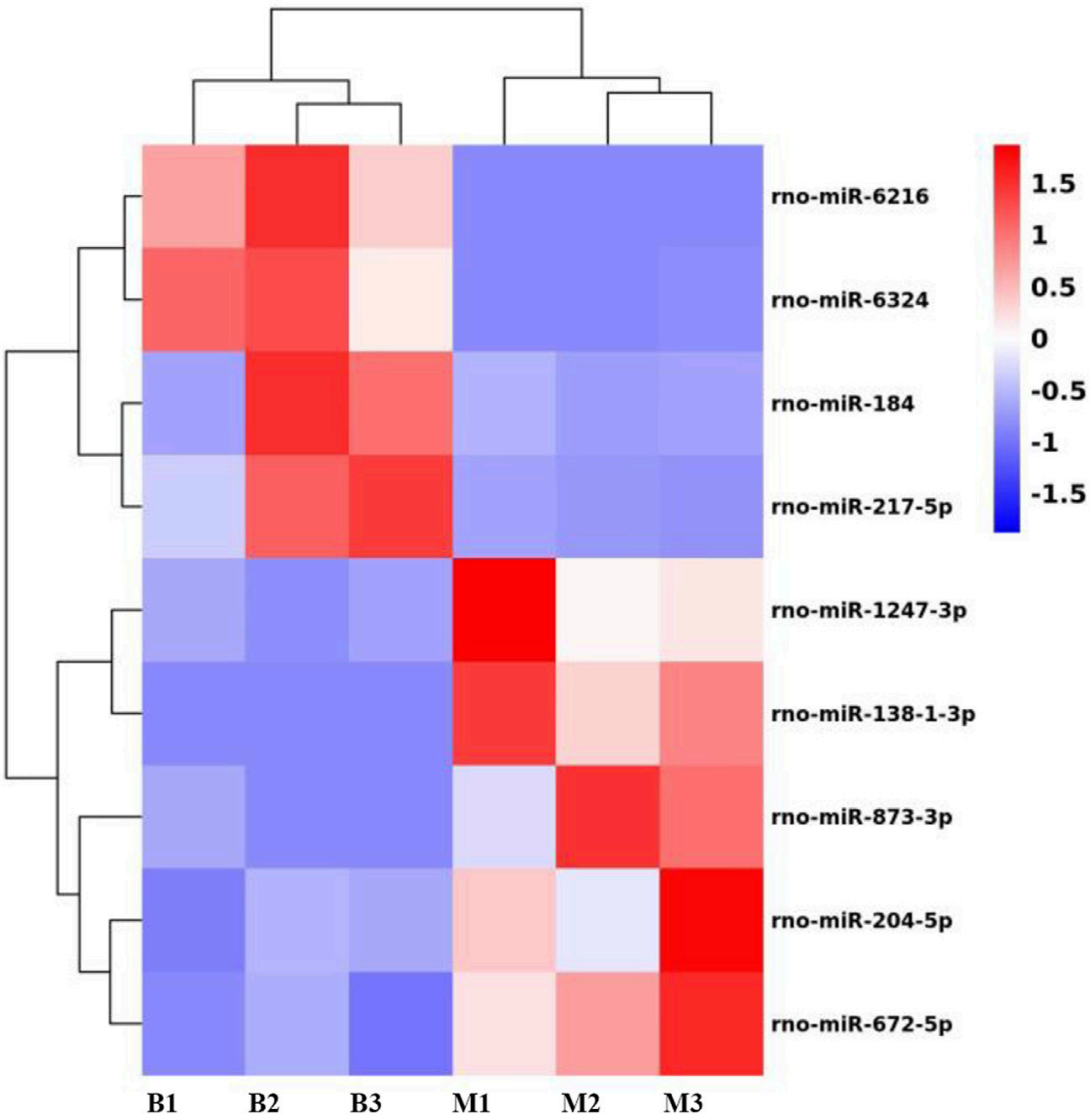
Heatmap results for the differential miRNAs. B in the horizontal axis indicates the blank group, M indicates the model group, and the number represents the number of each rat; the vertical axis indicates each differential gene. Red indicates upregulation of expression, blue indicates downregulation of expression.

After high-throughput gene sequencing and differential analysis, excluding the predicted genes, we made a volcano plot of the resulting genes, as shown in Figure 1. The left side shows the downregulated genes, there are four genes: miR-184, miR-6216, miR-6324, miR-217-5p; the right side shows the upregulated genes, there are five genes: miR-1247-3P, miR-138-1-3p, miR-204-5p, miR-672-5p, miR-873-3p. Meanwhile, we plotted the heatmap, as shown in Figure 2, and we labeled these nine differential genes above. B represents the blank group, M represents the model group, and the numbers represent the number of each group of rats, blue is downregulated, and red is upregulated. We performed subsequent KEGG and GO analysis of all the nine differential miRNAs and found that the downstream pathway of miR-6324 contains the neurotrophic signaling pathway, and several factors in this pathway are closely associated with the attack of IBS-D. Therefore, we chose miR-6324 as the key point of this study.

### 3.3 Gene function analysis

After target gene prediction and obtaining crossover genes for miR-6324 using two sites, miRWalk 3.0 and targetscan 7.2, we obtained 482 target genes. Then GO and KEGG analysis was performed based on these 482 target genes.

GO analysis (Gene Ontology) can be divided into three parts, namely, Biological Process (BP), Cellular Component (CC), and Molecular Function (MF) (Bai et al., 2020). Tables were obtained after GO analysis of the above 482 target genes using the DAVID website online, and data with *p*-values <0.05 were selected for retention. There are 55 results for GO-BP, including protein phosphorylation, positive regulation of transcription, positive regulation of cell proliferation, and intracellular signal transduction; There are 18 GO-CC results are affecting a variety of cellular components including cytoplasm, nucleus, cell membrane, and organelles on the intracellular surface; G0-MF has 22 results, mainly involved in protein binding, ATP binding, metal ion binding, DNA binding, and other molecular functions.

KEGG pathway enrichment analysis was performed on the above 482 target genes using the DAVID website, and data with *p*-values <0.05 were selected for backup preservation.15 pathways were enriched, mainly in cancer pathways, proteoglycans in cancer, neurotrophic signaling pathway, MAPK signaling pathway, RAP1 signaling pathway, and regulation of the actin cytoskeleton.

**FIGURE 3:**
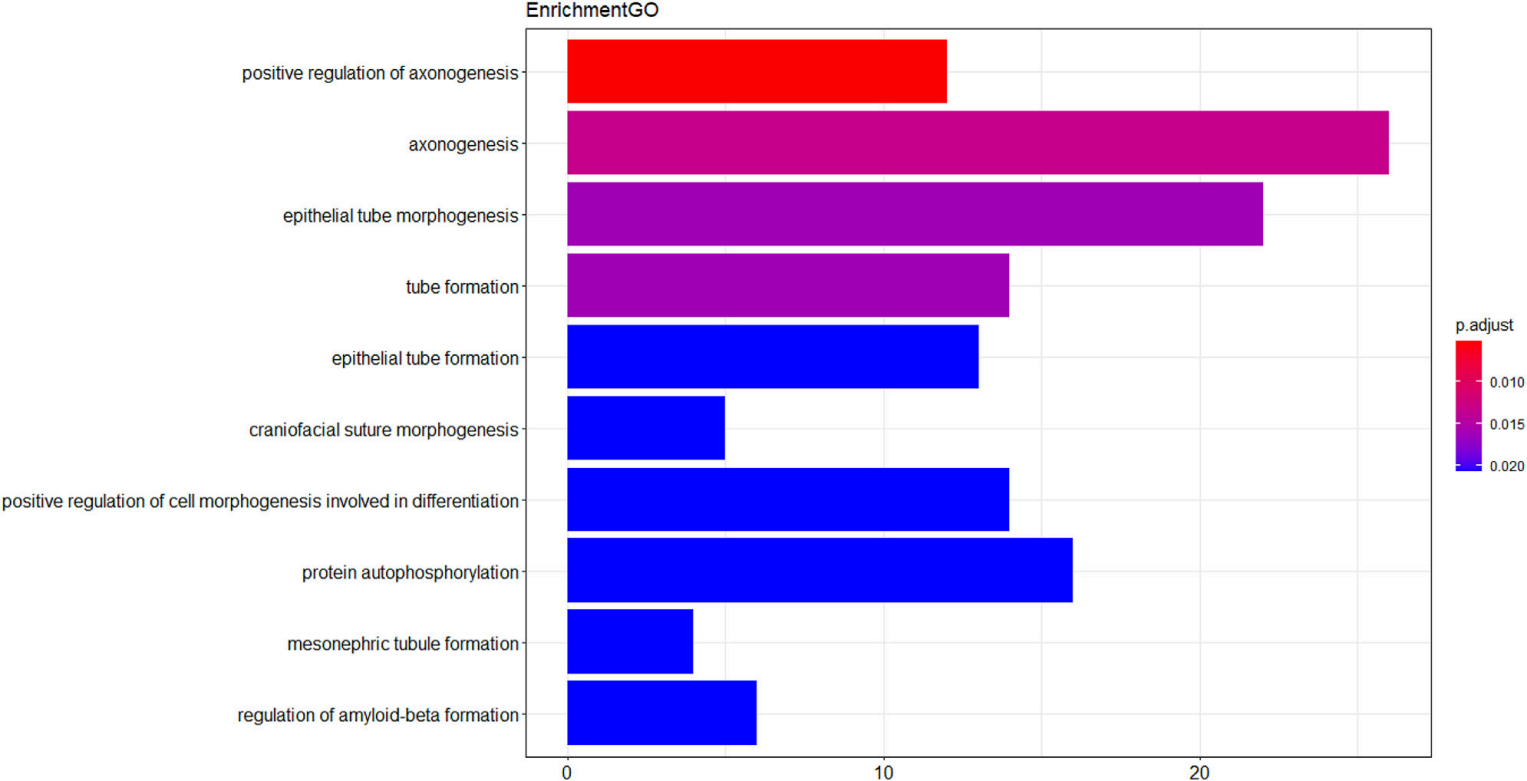
Histogram of GO analysis of intersecting target genes. The vertical axis is the entry name of Gene Ontology, the horizontal axis is the number of genes enriched in that entry, the closer the color is to red means the lower the *p*-value.

**FIGURE 4:**
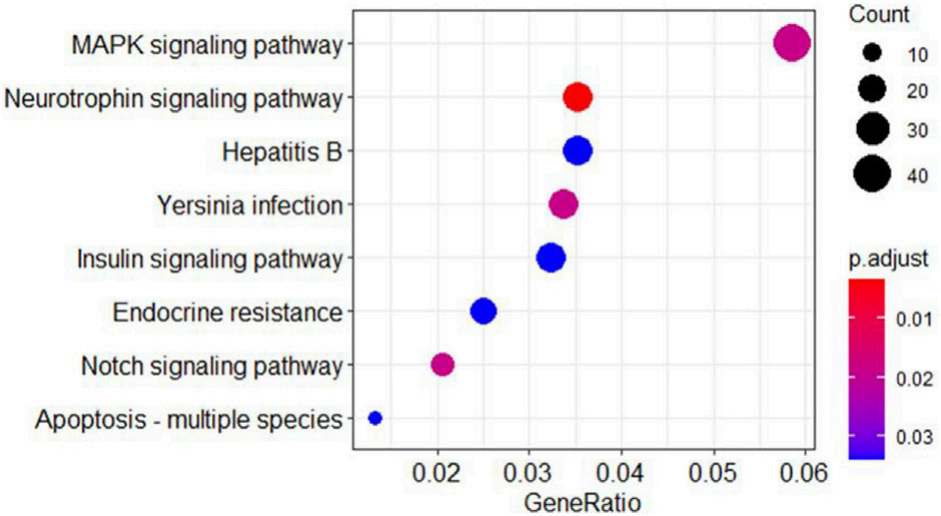
Bubble diagram for KEGG analysis of intersecting target genes. The horizontal axis indicates gene ratios and the vertical axis represent each pathway. Each bubble in the graph represents the pathway, with larger bubbles indicating more genes in the pathway and the color tending to red indicating smaller *p* values.

Gene GO analysis was performed on the 482 target genes obtained using RStudio software and the ClusterProfiler package as well as the DOSE package, the top 20 items were selected and made into a bar graph, as in Figure 3; for KEGG pathway analysis, the top eight were selected and made into a bubble graph, as in Figure 4.

### 3.4 protein-protein interaction (PPI) analysis

**FIGURE 5:**
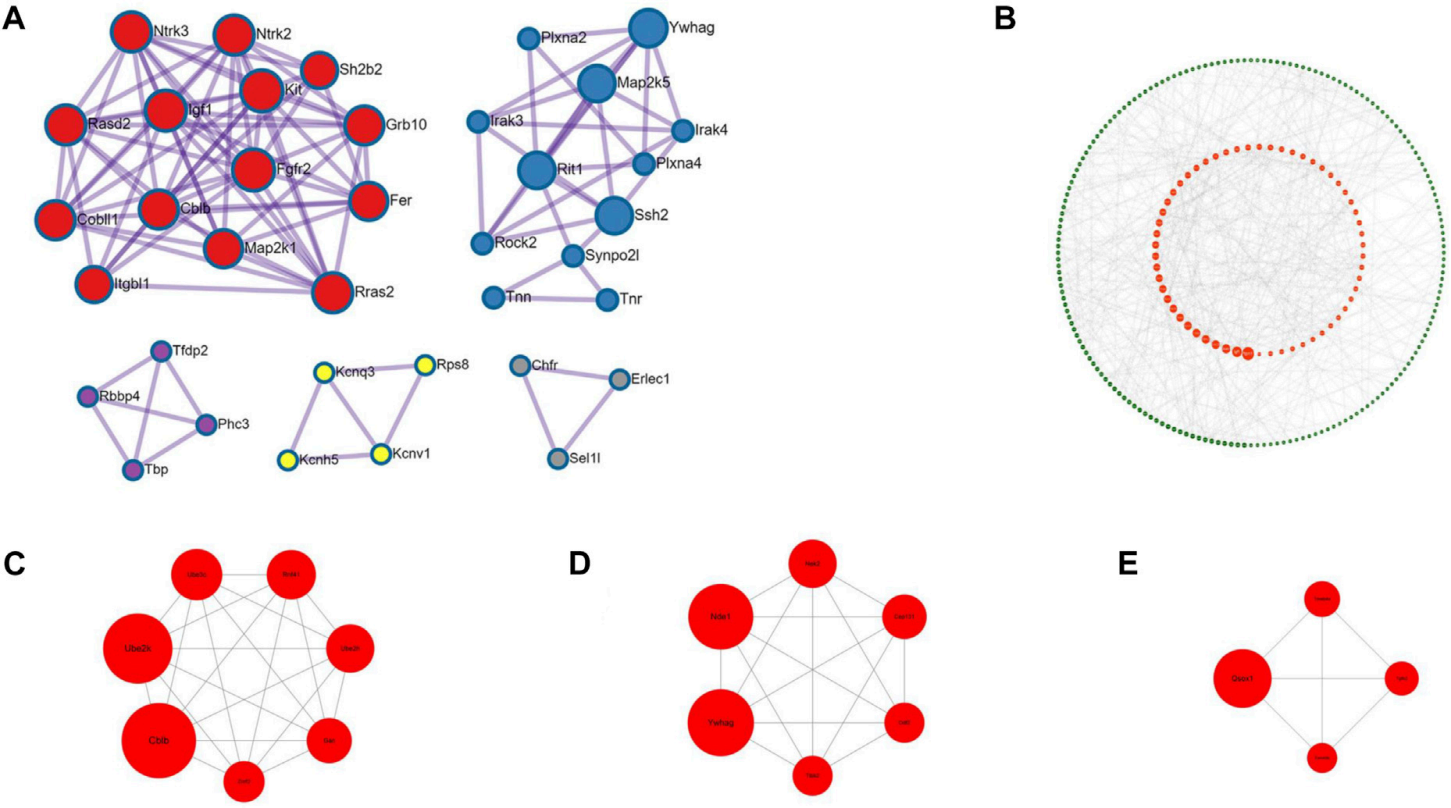
Protein functional classification chart and key proteins **(A)** is the protein functional clustering analysis graph, different colors represent different functional aggregation modules, the main modules are red and blue, the detailed description can be found in Table.3 **(B)** is all the proteins, sorted according to the BC value, the larger the dot in the graph represents the heavier weight, red is the top 1/4 of proteins with heavier weight **(C–E)** are the top three graphs filtered out, which include a total of 17 key genes.

As shown in Figure 5, the 482 target genes were analyzed using Cytoscape software to obtain the protein interaction network of the crossover genome. The protein function clustering analysis was also performed on these genes to obtain the protein function classification map, as shown in Figure 5A, From the figure, it can be seen that the protein functions are mainly clustered in two modules in red as well as blue, besides that, there are more functions, as shown in Table 3.

**TABLE 3:** Protein function module notes.

Color	MCODE	GO	Description	Log10(P)
Red	MCODE_1	R-MMU-9006934	Signaling by Receptor Tyrosine Kinases	−12.2
Red	MCODE_1	GO:0007169	Transmembrane receptor protein tyrosine kinase signaling pathway	−10.4
Red	MCODE_1	WP407	Kit receptor signaling pathway	−9.2
Blue	MCODE_2	GO:0032989	Cellular component morphogenesis	−6.1
Blue	MCODE_2	R-MMU-373755	Semaphorin interactions	−5.6
Blue	MCODE_2	mmu04360	Axon guidance-Mus musculus (house mouse)	−5.6
Purple	MCODE_3	R-MMU-212436	Generic Transcription Pathway	−3.7
Yellow	MCODE_4	GO:0071805	Potassium ion transmembrane transport	−5.9
Yellow	MCODE_4	GO:006813	Potassium ion transport	−5.7
Yellow	MCODE_4	GO:0098662	Inorganic cation transmembrane transport	−4.4
Grey	MCODE_5	GO:0051603	Proteolysis involved in protein process	−4.6
Grey	MCODE_5	GO:0030163	Protein catabolic process	−4.3

Then using the cytoNCA plug-in, the individual genes were weighted and sorted according to the Betweenness Centrality (BC), and then distributed as a circle, as shown is in Figure 5B. The larger graph of each gene in Figure 5B indicates the heavier weight, and the red circle in the center indicates the gene with the heavier 1/4 weight. The 482 target genes were analyzed using the MCODE plug-in, setting Find clusters = In whole network; Degree cutoff = 2; Node score cutoff = 0.2; K-core = 2; Max depth = 100 for analysis. In the generated network graph, the top three pairs were selected as the key genes in this study, 17 in total, as shown in Figures 5C, D, E, since some of the genes do not have clear functional studies for the time being, we cannot know whether all 17 genes are associated with the development of IBS-D, but by searching the literature we learned that the following genes were shown to be closely associated with the development of IBS-D, mainly Ube2k, Rnf41, Cblb, Nek2, Nde1, Cep131, Tgfb2, Qsox1, and Tmsb4x.

### 3.5 qPCR for miRNA

**FIGURE 6:**
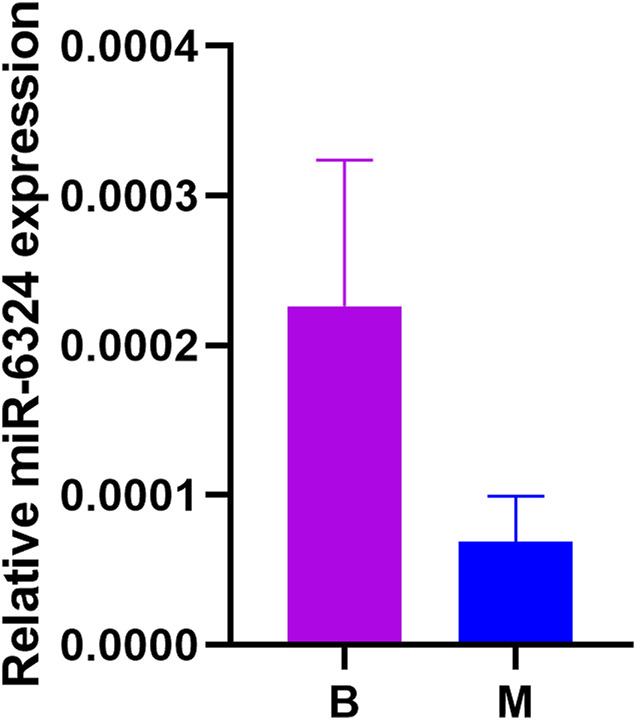
Relative miR-6324 expression. B in the horizontal axis means blank group, M means model group; the vertical axis represents the relative expression of miR-6324. *p* > 0.05 for the comparison between the two groups, no significant difference. However, there was a trend of decreasing expression in the model group.

After obtaining the relative expression levels of miR-6324, we analyzed the data of both groups and used Graphpad Prism eight to draw graphs. As shown in Figure 6, miR-6324 expression was decreased in the model group compared with the blank group, but not significantly (*p* > 0.05).

## 4 Conclusion

Since the discovery of microRNAs, their regulation of gene expression has received attention from various studies, and since the pathogenesis of irritable bowel syndrome is not clear, microRNAs in colonic tissues and irritable bowel syndrome have been studied more closely in recent years. Previous studies have shown that all subtypes of irritable bowel syndrome are inextricably linked to MicroRNA (Yang et al., 2020). This study demonstrated the correlation between miRNA-6324 and the development of IBS-D, which is valuable for the study of the pathogenesis of irritable bowel syndrome and further guides the medication through clinical trials.

In this study, the differential microRNAs obtained from the preliminary basic experiments were analyzed by bioinformatics. MiR-6324 was analyzed in the Kyoto Encyclopedia of Genes and Genomes database (KEGG), where the MAPK pathway (Guo et al., 2020a), the BDNF/TrkB pathway in the neurotrophic signaling pathway (Fu et al., 2021), and the PI3K pathway in the insulin signaling pathway (Xia et al., 2021) were shown to be associated with the pathogenesis of IBS-D in the preliminary study. In addition, for the core genes screened in the PPI network, Ube2k promotes the degradation of histone H3 (Fatima et al., 2020), and acetylation of histone H3 promotes visceral hypersensitivity in rats (Guo et al., 2020b), which is associated with the development of IBS-D; Rnf41 (Masschaele et al., 2017), Tmsb4x (Li et al., 2019a) mediates the pathogenesis of IBS-D by participating in the activation of the Myd88 pathway (Chu et al., 2020); the mammalian Cbl protein family has three members, Cbl, Cbl-b, and Cbl-3. Cbl-b regulates the levels of IFN-γ (Li et al., 2019b). IFN-γ is possibly involved in the pathogenesis of IBS-D after infection (He et al., 2018); Nek2 is involved in the pathogenesis of IBS-D by activating the AKT pathway (Wan et al., 2021) pathway (Xia et al., 2021); Nde1 is involved in the pathogenesis of IBS-D by regulating the MAPK pathway (Lanctot et al., 2013) (Xia et al., 2021); Cep131 (Wang et al., 2020), Tgfb2 (Guo et al., 2016), Qsox1 (Geng et al., 2020) mediate IBS-D pathogenesis through activation of PI3K/AKT pathway as well as other factors (Guo et al., 2020b). The above pathways as well as core genes are reflected in bioinformatic analysis to have an important relationship with the pathogenesis of IBS-D and are important components of the pathogenesis of IBS-D. At the same time, our qPCR experiment also verified that the expression of miR-6324 in the IBS-D model group decreased, which was the same as our previous high-throughput sequencing results. The research above proves that the pathogenesis of IBS-D is regulated by miR-6324, and further in-depth studies are needed as to which way it mediates the pathogenesis and which role it plays in the pathogenesis of IBS-D.

Studies have shown that several microRNAs, including miR-29 (Zhou et al., 2015), miR-199b (Mansour et al., 2016), miR-219a-5p and miR-338-3p (Mahurkar-Joshi et al., 2021), and miR-29a (Chao et al., 2017), have important roles in the pathogenesis of diarrheal irritable bowel syndrome. There are no studies available to demonstrate the close correlation between miR-6324 and IBS-D. We reviewed some literature on miR-6324 and one study found that inhibition of miR-6324 expression was associated with rescue of cell viability, inhibition of apoptosis, and promotion of autophagy (Li et al., 2021). Our volcano, heat maps, and qPCR result showed that miR-6324 expression was downregulated in the model group, which is contrary to the above findings. We reviewed the relevant literature and one study demonstrated that miRNAs were differentially expressed in different tissues, whereas miR-6324 was expressed higher in brain from the ectoderm than in other tissues and higher in the hippocampus (Cheng et al., 2021). Our previous study found that BDNF mRNA expression was significantly downregulated in rat colonic tissue and significantly upregulated in hippocampal tissue after IBS-D modeling. Meanwhile, we demonstrated that tong-xie-yao-fang can downregulate hippocampal BDNF mRNA expression and upregulate colonic BDNF mRNA expression, which is a bidirectional regulation (Chen et al., 2017). Based on our previous study, we suggest that miR-6324 expression may be elevated in the hippocampus of IBS-D model rats in this study, which is also a bidirectional regulation.

Since the association between the brain-gut axis and IBS-D has been demonstrated in several studies, we speculated that it is possible that miR-6324 expression is elevated in the hippocampus and decreased in the colon in the IBS-D rat model. Although the present study on miR-6324 has only demonstrated the association with heart infarction as well as hippocampal nerves, we believe that our study is meaningful and has some prospects for development, which may somewhat reveal the mechanism related to miRNA regulation of IBS-D through the brain-gut axis and provide ideas for further studies to follow.

In this study, we detected a significant decrease in miR-6324 expression in rat colon tissue in basic experiments and revealed its possible involvement in the pathogenesis of IBS-D utilizing bioinformatic analysis. Finally, we performed qPCR experiments on miR-6324, and although we did not conclude that miR-6324 was significantly reduced in the IBS-D model, we obtained a decreasing trend. We believe that the insignificant decrease in expression may be related to the small sample size and the degradation of RNA, which is a shortcoming of this experiment. Based on our speculation and the studies of others, our next study will focus on whether there are differences in the expression of miR-6324 or other miRNAs in colonic and hippocampal tissues in the brain-gut axis and further investigate the association of the brain-gut axis with IBS-D.

## Data Availability

The datasets generated for this study can be found in the GEO database, accession no. GSE222757 (https://www.ncbi.nlm.nih.gov/geo/query/acc.cgi?acc=GSE222757).
