# Corrigendum: Study on the pathogenesis of MiR-6324 regulating diarrheal irritable bowel syndrome and bioinformatics analysis

**DOI:** 10.3389/fphar.2023.1330698

**Published:** 2023-12-07

**Authors:** Jin Xiao, Yan-ni Zhou, Yan-lin Yang, Li He, Ke-kai Wang, Min Chen

**Affiliations:** ^1^ Hospital of Chengdu University of Traditional Chinese Medicine, Chengdu, Sichuan, China; ^2^ Sichuan Hospital of Integrative Medicine TCM, Chengdu, Sichuan, China; ^3^ Zigong Fifth People’s Hospital, Zigong, Sichuan, China; ^4^ Traditional Chinese Medicine Hospital, Chongqing, China

**Keywords:** miR-6324, irritable bowel syndrome, bioinformatics analysis, pathogenesis, R language

In the published article, there was an error in **Affiliation** for author Chen Min. Instead of “Chengdu University of Chinese Medicine School of Clinical Medicine, Sichuan Provincial Hospital of TCM, Chengdu, Sichuan, China,” their affiliation should be “Hospital of Chengdu University of Traditional Chinese Medicine, Chengdu, Sichuan, China.”

The authors apologize for this error and state that this does not change the scientific conclusions of the article in any way. The original article has been updated.

