# Supplementary figures and images for: Study on the pathogenesis of MiR-6324 regulating diarrheal irritable bowel syndrome and bioinformatics analysis
Source: Front Pharmacol. 2023 Feb 15;14:1044330. doi: 10.3389/fphar.2023.1044330 (PMC9975503; doi:10.3389/fphar.2023.1044330)

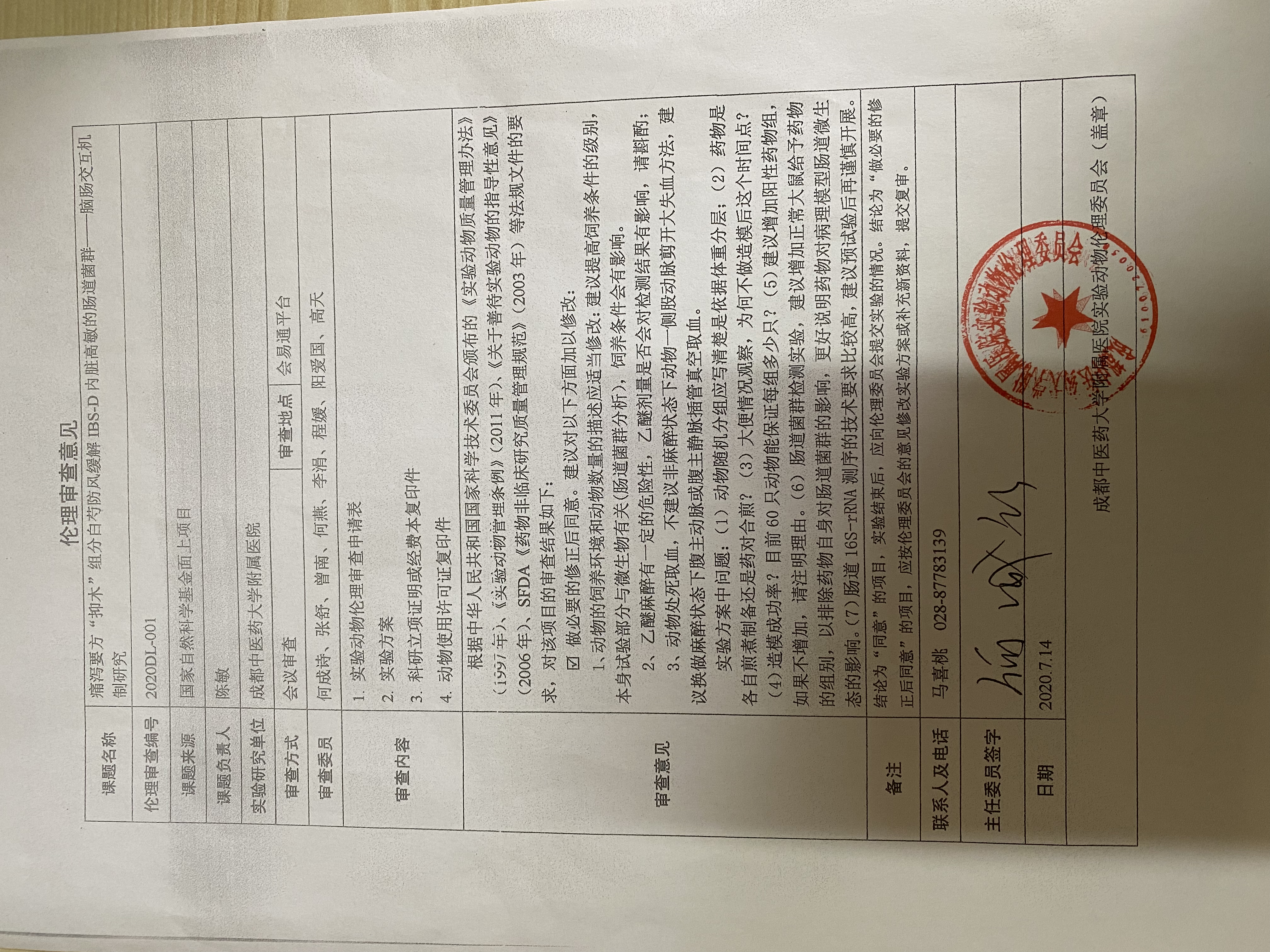

Supplement: Supplementary file 1 [file Image1.JPEG]
